# POWER Up—Improving pre-exposure prophylaxis (PrEP) uptake among Black cisgender women in the Southern United States: Protocol for a stepped-wedge cluster randomized trial (SW-CRT)

**DOI:** 10.1371/journal.pone.0285858

**Published:** 2023-05-17

**Authors:** Jessica P. Ridgway, Samantha A. Devlin, Eleanor E. Friedman, Ososese E. Enaholo, Maria Pyra, Lisa R. Hirschhorn, Sadia Haider, Kelly Ducheny, Amy K. Johnson

**Affiliations:** 1 Department of Medicine, Section of Infectious Diseases and Global Health, University of Chicago, Chicago, IL, United States of America; 2 The Potocsnak Family Division of Adolescent and Young Adult Medicine, Ann & Robert H. Lurie Children’s Hospital, Chicago, IL, United States of America; 3 Department of Medical Social Sciences, Feinberg School of Medicine, Northwestern University, Chicago, IL, United States of America; 4 Division of Family Planning, Rush University, Chicago, IL, United States of America; 5 Howard Brown Health, Chicago, IL, United States of America; GERMANY

## Abstract

**Background:**

HIV disproportionately affects Black/African American cisgender women (hereafter women) in the United States. Despite its proven effectiveness, pre-exposure prophylaxis (PrEP) for HIV prevention remains vastly under-prescribed to women based on their need. Increasing PrEP uptake and persistence among women is crucial to reducing HIV transmission; however, there have been few studies designed specifically for women. This article describes the study protocol used to assess the feasibility, acceptability, and effectiveness of implementation strategies to improve PrEP uptake and persistence among Black women in the Midwest and South.

**Methods:**

PrEP Optimization among Women to Enhance Retention and Uptake (POWER Up) is an evidence-based, woman-focused set of five implementation science strategies that addresses barriers of PrEP utilization at the provider, patient, and clinic levels. POWER Up includes 1) routine PrEP education for patients, 2) standardized provider training, 3) electronic medical record (EMR) optimization, 4) PrEP navigation, and 5) PrEP clinical champions. These strategies will be adapted to specific clinics for implementation, tested via a stepped-wedge trial, and, if effective, packaged for further dissemination.

**Discussion:**

We will utilize a stepped-wedge cluster randomized trial (SW-CRT) to measure change in PrEP utilization across diverse geographic areas. Preparation for adapting and implementing the bundle of strategies is needed to determine how to tailor them to specific clinics. Implementation challenges will include adapting strategies with the available resources at each site, maintaining stakeholder involvement and staff buy-in, adjusting the study protocol and planned procedures as needed, and ensuring minimal crossover. Additionally, strengths and limitations of each strategy must be examined before, during, and after the adaptation and implementation processes. Finally, the implementation outcomes of the strategies must be evaluated to determine the real-world success of the strategies. This study is an important step toward addressing the inequity in PrEP service delivery and increasing PrEP utilization among Black women in the U.S.

## Background

### A focus on women and under-prescription of PrEP

Black/African American cisgender women (hereafter women) experience one of the highest HIV incidence rates among all subpopulations in the United States, second only to men who have sex with men (MSM). Despite constituting only 13% of the population, Black women account for 60% of new HIV infections among women in the U.S [1]. Moreover, Black women are disproportionately affected by HIV, with an 18-fold higher risk of acquiring HIV compared to white women [2].

In spite of a high HIV incidence, use of pre-exposure prophylaxis (PrEP) among Black women is very low. PrEP is highly effective for HIV prevention [3], but women make up a disproportionately low percentage of PrEP users compared to men. In 2021, the PrEP-to-need ratio (defined as number of PrEP prescriptions divided by number of new HIV diagnoses) for women was substantially lower than that for men (4.1 vs 11.2), indicating an inequity in PrEP use among women with respect to their need [4]. Black women in particular are underrepresented among PrEP users [5, 6]. Furthermore, among individuals who initiate PrEP, both women and Black patients are less likely to persist on PrEP compared to their white male counterparts [7, 8].

In addition to racial disparities in HIV incidence and PrEP use among women, prominent geographic disparities also exist. The South represents the greatest burden of HIV in the U.S. and ranks lower than other regions in terms of HIV prevention implementation. The majority of women who acquire HIV in the U.S. live in the South [2]. Although the region has more than half of new annual HIV cases, Southerners accounted for only 39% of all PrEP users in 2021 [9]. Additionally, women in the Midwest (4.57) and South (4.87) had the lowest PrEP-to-need ratios (indicating the highest unmet need for PrEP) among any subgroup in the U.S in 2021 [9]. Improving access to effective HIV prevention strategies for Black women in the Southern U.S. is critical to address these racial and geographic disparities and to eliminate HIV transmission in the United States.

### Addressing the barriers to PrEP initiation and persistence among women

There are several individual-, provider-, and clinic-level challenges to PrEP initiation and persistence for Black women [10]. PrEP awareness is limited in the community [11, 12], and Black women often have low self-perceived risk for HIV acquisition despite having certain risk factors; moreover, these risk factors among women were generally overlooked by previous guidelines for prescribing PrEP [13–15]. The Centers for Disease Control and Prevention (CDC) revised their PrEP guidelines in December 2021 to recommend that healthcare providers give information about PrEP to all sexually active adults and adolescents and to recommend PrEP for all adults and adolescents with risks for HIV exposure [16]. However, a significant proportion of medical providers do not routinely discuss PrEP or prescribe PrEP for their female patients [17, 18]. Indeed, most Black women would prefer to receive PrEP from trusted primary care providers [11], and the majority of women newly diagnosed with HIV attended medical visits prior to their HIV diagnosis, representing missed opportunities by medical providers for initiating PrEP [19]. Additionally, structural and social barriers exist to both PrEP initiation and persistence, including cost, healthcare access, stigma, lack of PrEP awareness/familiarity among patients, and comorbid substance use or mental health disorders [20–22]. Although there has been growing knowledge of ways to increase PrEP use by MSM and transgender women [23–27], much less is known about successful strategies to increase PrEP uptake and support PrEP persistence among Black women.

### Implementation of POWER Up

**Fig 1:**
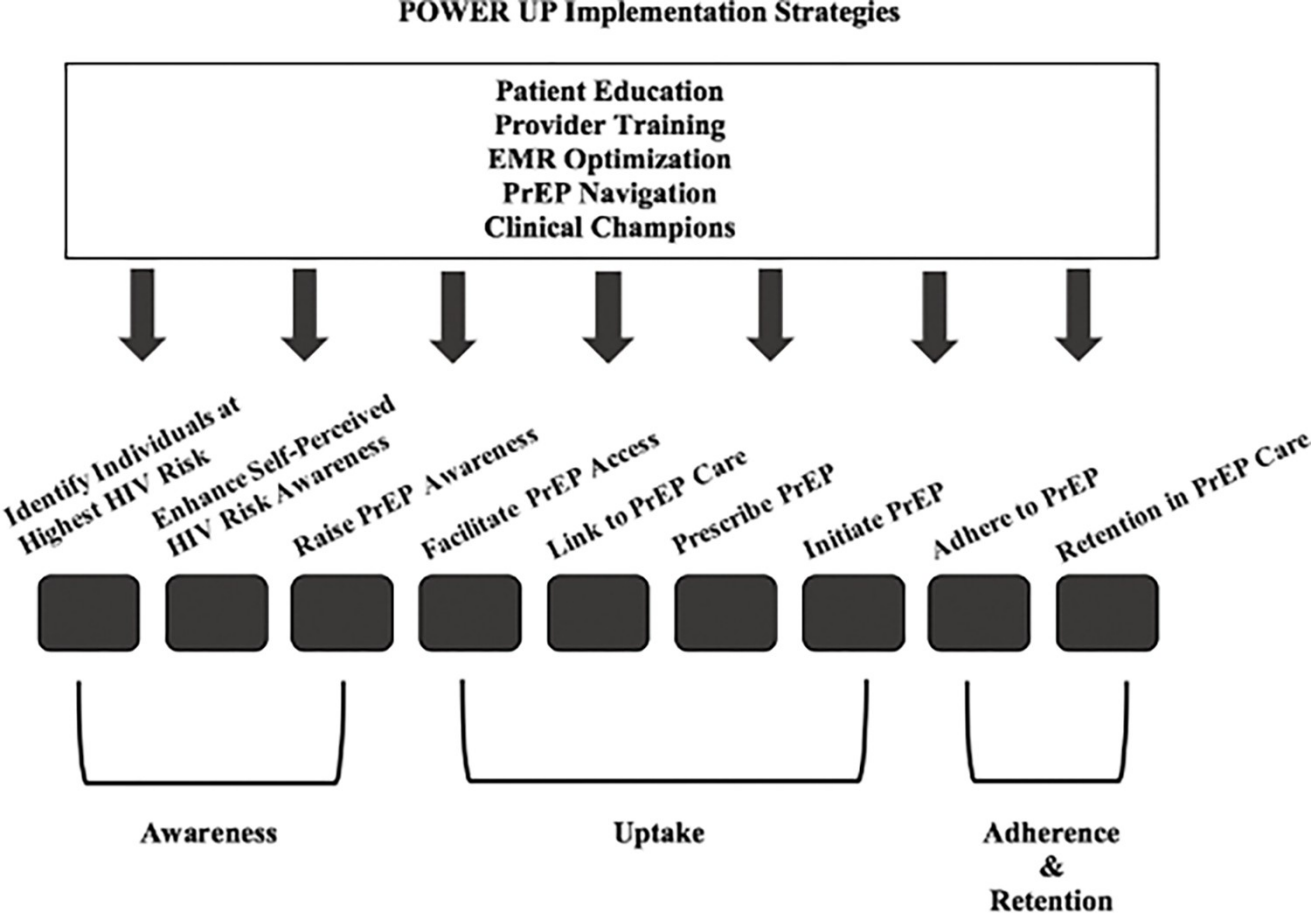
Effects of the POWER Up implementation strategies on the PrEP care continuum.

To improve PrEP uptake and persistence among Black women, multifaceted and multi-level strategies must target each step of the PrEP care continuum (i.e., awareness of PrEP, identification of PrEP-eligible candidates, prescription and initiation of PrEP, persistence of care during HIV risk periods, and re-initiation of care following discontinuance) [10]. Through preliminary work, our team has identified successful strategies for improving PrEP care continuum outcomes among Black women. We performed formative research guided by the Consolidated Framework for Implementation Research (CFIR 2.0) [28], including mixed methods studies of client-, health system-, and provider-level barriers and facilitators to PrEP initiation and persistence among Black women [11, 29–33]. Through this work, we have identified a set of five strategies that have been successful in community health clinic (CHC) settings for PrEP Optimization among Women to Enhance Retention and Uptake (POWER Up). POWER Up strategies include 1) routine PrEP education to raise awareness of PrEP among Black women; 2) standardized provider training for how to identify women who are PrEP candidates, discuss HIV risk and teach women about PrEP, and prescribe PrEP and engage them in PrEP care; 3) electronic medical record (EMR) tools for identifying women who would benefit from PrEP, prescribing PrEP, and tracking PrEP outcomes; 4) PrEP navigation to assist with PrEP initiation and persistence; and 5) clinical champions for supporting the successful creation and implementation of PrEP-related processes (Fig 1). With these POWER Up strategies, a CHC in the Midwest has more than doubled the rate of PrEP uptake among Black women and maintained PrEP persistence rates above the national average (mean persistence among ciswomen of 8.1 months vs. 5.8 months nationally) [7, 29]. Our study will implement this bundle of strategies in 3 CHC networks (1 in the Midwest and 2 in the South), with the goal of increasing PrEP uptake and persistence among Black women.

We hypothesize the following effects: 1) women at the CHCs will demonstrate a clinically meaningful increase in PrEP uptake and persistence at 3 and 6-months post-baseline, with 10% of PrEP-eligible Black women initiating PrEP and 30% of women who initiate PrEP persisting, and 2) the implementation of these strategies will be fully adopted by ≥80% of clinic partners and will achieve ≥ 90% ratings of satisfaction and acceptability by clinical partners and patients, and PrEP eligibility assessment will reach ≥ 50% of Black women.

## Materials and methods

### Aims and objectives

The aim of this study is to assess the acceptability and feasibility of implementing POWER Up as part of routine care in CHC settings and to assess the adaptation and efficacy of the implementation strategy package as it is implemented by comparing pre- and post-implementation rates of PrEP prescription among Black women at each CHC. The project has three specific aims. Aim 1 is to undertake qualitative data collection through provider and patient focus group discussions (FGDs) to inform the adaptation and implementation of POWER Up strategies for engaging Black women in the PrEP care continuum for use in CHCs. Aim 2 is to measure the implementation and effectiveness outcomes of the adapted bundle of implementation strategies in the Southern United States compared to the approach in the Midwest. This aim will be accomplished via a stepped-wedge cluster randomized trial (SW-CRT) to measure change in implementation outcomes and evaluation using the Reach, Effectiveness, Adoption, Implementation, and Maintenance (RE-AIM) framework. We will implement the POWER Up strategies across diverse geographic areas and assess the change in clinical and implementation outcomes from the pre-implementation (control) and the post-implementation (implementation) periods. The cluster, or unit of analysis, is each CHC, N = 12 (8 in the South, 4 in the Midwest). Aim 3 is to develop an implementation guide that can be utilized across U.S. jurisdictions to improve PrEP care continuum engagement among Black women, reflecting the knowledge gained through the adaptation and evaluation processes.

### Settings

POWER Up is being rolled out sequentially in 12 CHCs among 3 health networks in the Southern and Midwestern United States: Advance Community Health (North Carolina), Evara Heath (Pinellas County, Florida) and Erie Family Health Centers (Cook County, Illinois). Two of the health networks are located within priority jurisdictions of the CDC’s Ending the HIV Epidemic (EHE) initiative based on the high burden of HIV infection (Pinellas County, FL and Cook County, IL) [34]. These networks were selected due to their area’s elevated incidence of HIV, as well as the high volume of PrEP-eligible Black women who receive care at their sites. POWER Up has not been replicated elsewhere, so it is important to consider geographic location as a factor in implementation success. Although the goal of this project is to improve PrEP care continuum outcomes among Black women in the South, we are also including Midwestern sites to serve as a comparison in effectiveness to Southern sites. We chose to include different geographic regions so that the dissemination guide is relevant nationally to increase the likelihood that POWER Up strategies are able to be implemented nationwide. All participating CHCs currently have very low PrEP usage among Black women (less than 1%) who receive care at their sites, and none of the CHC sites have previously implemented the bundle of POWER Up strategies.

### Study design

We will utilize a SW-CRT to measure change in our primary implementation outcome, PrEP uptake. This study design will allow us to roll out the bundle of implementation strategies across diverse geographic areas and to assess the change in clinical and implementation outcomes from the pre-implementation (control) and the post-implementation (implementation) periods. There will be 12 clusters: 8 CHCs in the South and 4 CHCs in the Midwest (N = 12). In a stepped-wedge study, each clinic contributes both time and observations to the control condition (usual care) and the implementation condition (implementation of POWER Up strategies). Every clinic switches from the control to the implementation at regular time intervals (steps), but at different points in calendar time depending on randomization order [35]. Additionally, more than one clinic may start the bundle of POWER Up implementation strategies at a step. The design uses repeated cross-sectional collection of data, with our primary outcome obtained from patients seen during each time period (each step) at each clinic rather than following individually recruited patients over time. For our two PrEP persistence measures, patients are followed longitudinally after starting PrEP. This is a robust design because each clinic provides data under both the control and implementation conditions, reducing variability between the two conditions and improving precision when intra-cluster correlation is high, which is likely among the CHCs that we have chosen. As stepped-wedge designs consist of a unidirectional move from control to implementation, the bundle of POWER Up strategies is not withdrawn once implemented, alleviating ethical concerns [36].

**Fig 2:**
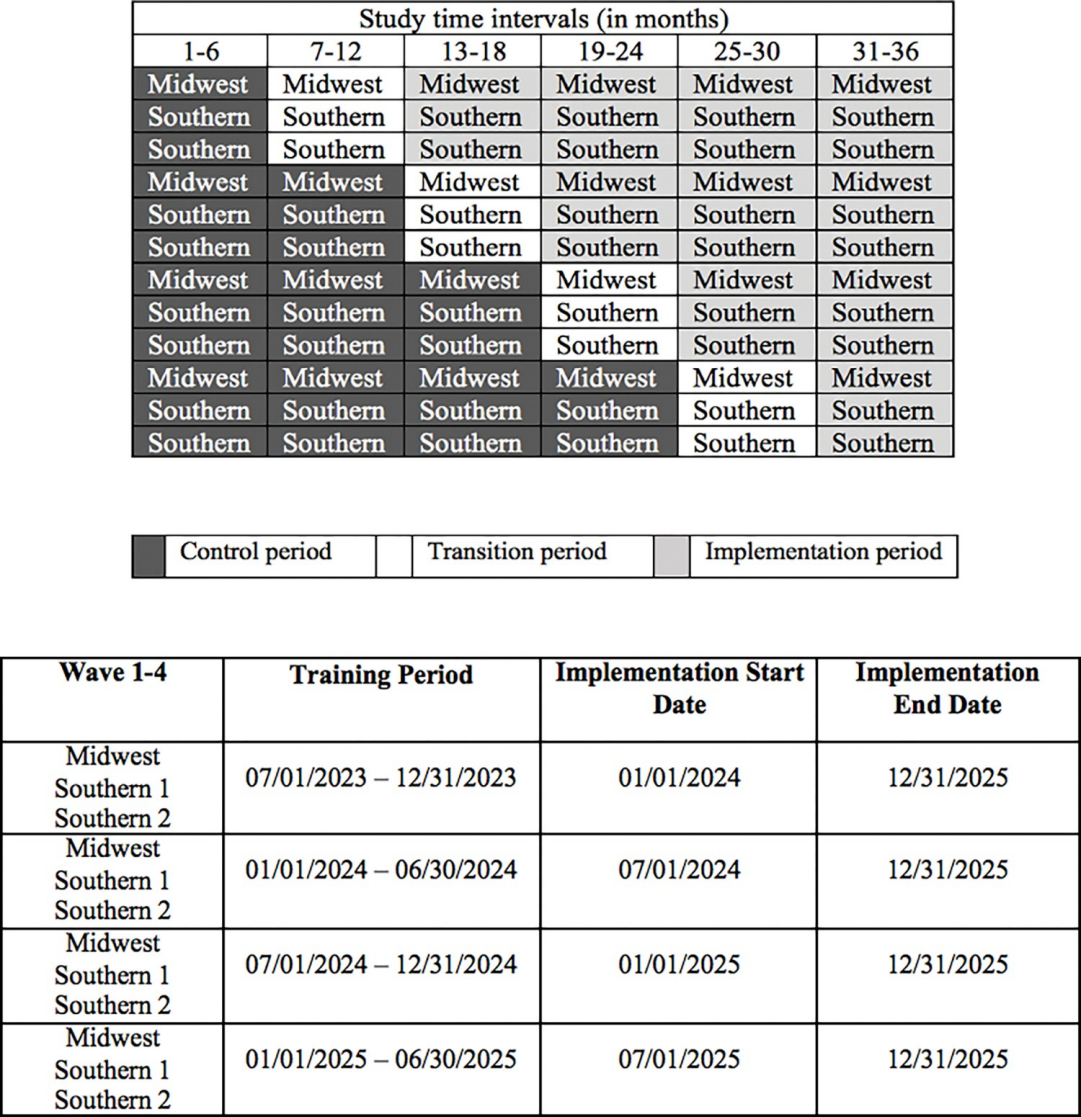
Timeline of stepped-wedge cluster randomized trial (SW-CRT) design (control, transition, and implementation periods).

In our stepped-wedge design, each step is 6 months long, and there are 4 waves of implementation (Fig 2). Three CHC sites will be randomized to each step, with a mix of sites from the South and the Midwest included. Each cluster also undergoes a 6-month transition period when the individual clinics will prepare to implement the bundle of POWER Up strategies. The sequence of switching from control to implementation condition at each site (each cluster) will be determined using a computer-based list randomization application to create 4 waves of implementation (three sites begin the implementation at the same step, a mix from the South and the Midwest).

### Study procedures

#### Data collection: Pre-implementation phase

The pre-implementation phase will occur from September 2021 to June 2023. During this phase, we will engage our expert advisory board–a group of medical providers, public health scientists specializing in PrEP implementation research, and Black women who have experience with HIV/PrEP. We will identify and engage with clinical champions at each health network to assist in the implementation design and provide feedback on local implementation. We will also conduct focus groups with providers and Black women patients at each of the clinic sites. Providers will be recruited using convenience sampling via email invitations at each clinic. Providers will respond to the email if interested in study participation. Flyers with the study team’s contact information will also be distributed to providers. Providers will be eligible to participate if they are 1) aged 18 years or older, 2) currently employed at one of the participating health network sites, 3) able to speak and understand English, and 4) provide informed verbal consent. Patients will be recruited at each CHC through flyers and routine phone calls made by staff, who will inform patients about the study. CHC staff will provide interested patients with the study team’s contact information, and patients will initiate contact with the study team. We will also provide a QR code on the flyer that will take patients to a study information page and eligibility screening form hosted on the University of Chicago’s secure web-based application, Research Electronic Data Capture (REDCap). The landing page will provide information about the focus groups, and patients will be able to enter their name and contact information so that the study team can screen patients for eligibility and contact them if eligible. To supplement passive recruitment by CHC staff, the study team will actively recruit in-person as needed at the study sites and conduct in-person eligibility screenings and focus groups. Patients will be eligible to participate if they 1) are aged 18 years or older, 2) are a current patient at one of the participating health network sites, 3) are able to speak and understand English, 4) identify as a Black/African American cisgender woman, and 5) provide informed verbal consent. Providers will be asked about PrEP training/education, existing PrEP education (e.g., brochures, flyers) available at their clinic, contextual factors that may inform adaptation of implementation materials, and workflow/approach to patient care (e.g., on-site pharmacy, blood draws available on site). The patient FGD guide will assess patients’ knowledge of and experience with PrEP, thoughts on PrEP education and being offered PrEP, suggestions for PrEP navigation services, and contextual factors influencing clinic environment and decision making about PrEP. After conducting focus groups and analyzing the qualitative data, we will use our findings to create relevant adaptations for each site (i.e., tailored provider training curricula and patient education materials/visuals).

#### Data collection: Implementation phase

In preparation for the implementation phase, we created a table of desired EMR variables that will be collected from each CHC throughout the implementation period (Table 1). These variables will be collected from July 1, 2023 to December 31, 2025. We discussed data specifications (e.g., demographic characteristics, PrEP prescriptions, sexually transmitted infections (STI) diagnoses, etc.) with site leaders and adapted the list of requested variables based on availability of elements within the EMR, the type of EMR used, and workflow of each CHC.

**Table 1:** Electronic medical record (EMR) data collected monthly during the stepped-wedge cluster randomized trial (SW-CRT).

*Primary Outcome*		
**Data Variable**	**Brief Description**	**RE-AIM Dimension**
PrEP uptake	Number of patients initiating PrEP	Effectiveness
*Secondary Outcomes*		
**Data Variable**	**Brief Description**	**RE-AIM Dimension**
Total patients eligible to learn more about PrEP	All patients eligible to learn more about PrEP according to study criteria. All patients 18 years or older, who are not HIV positive who have a medical encounter during which PrEP could be prescribed.	Reach
Demographics/clinical factors of total patients eligible to learn more about PrEP	Demographic data for patients eligible to learn more about PrEP.^a^	Reach
HIV risk assessment	Proportion of patients who undergo HIV risk assessment at each site	Reach
Demographics/clinical factors of patients receiving HIV risk assessment	Demographic data for patients who undergo HIV risk assessment.^a^	Reach
PrEP eligibility	Proportion of patients identified as PrEP eligible at each site.	Reach
Demographics/clinical factors of PrEP eligible patients	Demographic data for patients identified as PrEP eligible.^a^	Reach
Number of providers who educate patients about PrEP	Number of providers who offer PrEP education	Reach
Number of providers who prescribe PrEP	Number of providers who prescribe PrEP to participants	Reach
PrEP education	Proportion of patients who received PrEP education.	Reach
Demographics/clinical factors of PrEP educated patients	Demographic of patients who received PrEP education.^a^	Reach
PrEP offered	Proportion of patients offered PrEP.	Reach
Demographics/clinical factors of patients offered PrEP	Demographics of patients offered PrEP.^a^	Reach
PrEP navigation	Number of patients receiving PrEP navigation services	Reach
Demographics/clinical factors of PrEP navigated patients	Demographics of patients receiving PrEP navigation services.^a^	Reach
Demographics/clinical factors of patients who uptake PrEP	Demographics of patients initiating PrEP.^a^	Effectiveness
Number of providers receiving feedback	Number of CHC providers receiving feedback regarding their PrEP prescription rates	Effectiveness
PrEP ineligibility	Proportion of patients previously identified as PrEP eligible who are ineligible medically to start PrEP.	Effectiveness
Demographics/clinical factors of PrEP ineligible patients	Proportion of patients identified as PrEP ineligible.^a^	Effectiveness
PrEP persistence: Total time on PrEP	Proportion of patients who achieve 6-month persistence on PrEP. Total time on PrEP is measured from date of first prescription to end of last prescription.	Maintenance
Demographics/clinical factors of patients who meet the PrEP persistence Total time on PrEP measure	Demographics of patients who achieve 6-month persistence on PrEP.^a^	Maintenance
PrEP persistence: Proportion of days covered	Proportion of patients who achieve 6-month persistence on PrEP at each site. Patients are considered persistent if they have received a PrEP prescription to cover 6/7 days for each week within a given time period.	Maintenance
Demographics/clinical factors of patients who meet the PrEP persistence: Proportion of days covered measure	Demographics of patients who achieve PrEP persistence via the proportion of days covered measure.^a^	Maintenance

PrEP: Pre-exposure Prophylaxis; STI: Sexually Transmitted Infection; CHC: Community Health Clinic

^a^stratified by race, sex, ethnicity, age group, current gender, sexual identity, current medical complaint, and STI testing history in last year

We will collect both patient-level and clinic-level data from each CHC on a monthly basis. These data will be collected in two ways: 1) by the aggregation of data from patient-level EMR records and 2) by surveys among patients and providers designed to measure knowledge, beliefs, and satisfaction regarding PrEP and the implementation strategies. All EMR data will be collected during each of the three phases of the stepped-wedge trial: 1) pre-implementation; 2) transition, and 3) implementation phases. Table 1 shows the list of EMR variables that will be collected from each site on a monthly basis during the stepped-wedge trial. To obtain these EMR variables, we will aggregate data from individual patient records (~3,000) that meet each variable definition. No recruitment of patients is required for the monthly EMR data collection, as POWER Up is considered part of routine care. The survey data that will be collected vary in the stages of the stepped-wedge trial and the frequency of assessment. Some of these measures will be collected and evaluated monthly, and some will be collected once for pre- and post-implementation (i.e., pre-implementation and at the end of the implementation period). Clinical champions at each site will assist with monitoring adherence to implementation protocols at each site. Table 2 shows outcomes that will be collected via quantitative surveys and qualitative interviews based on the RE-AIM framework [37] and their associated time periods of collection. These surveys and interview guides will be composed of previously validated questions whenever possible and include CFIR domains to assess implementation success. CHC providers and patients will be recruited to complete these quantitative surveys and qualitative interviews via the same recruitment methods used for pre-implementation focus groups (e.g., provider recruitment emails and routine phone calls/recruitment flyers for patients).

**Table 2:** Data collected for evaluation of implementation using the RE-AIM framework.

Data Variable	Time Collected from Healthcare Network	Brief Description & Source of Data	RE-AIM Dimension
Change in provider knowledge and attitudes about PrEP	Once during control period (baseline) and once at end of implementation period	Average change in score between baseline and post implementation survey about provider knowledge and attitudes about PrEP; qualitative interview	Effectiveness
Change in patient knowledge and attitudes about PrEP	Once during control period (baseline) and once at end of implementation period	Average change in score between baseline and post implementation survey about patient knowledge and attitudes about PrEP; qualitative interview	Effectiveness
Number of providers who complete training	Once after provider training	Number of providers who complete online training	Adoption
Acceptability for providers	Once at end of implementation	Average score among providers of the Acceptability of Intervention Measure tool (AIM); qualitative interviews	Implementation
Acceptability for patients	Once at end of implementation	Average score among patients of the Acceptability of Intervention Measure tool (AIM); qualitative interviews	Implementation
Appropriateness of implementation for providers	Once at end of implementation	Average score among providers of the Intervention Appropriateness Measure tool (IAM); qualitative interviews	Implementation
Appropriateness of implementation for patients	Once at end of implementation	Average score among patients Intervention Appropriateness Measure tool (IAM); qualitative interviews	Implementation
Maintenance of implementation for providers	Once at end of implementation	Average score among providers of the Program Sustainability Assessment Tool (PSAT); qualitative interviews	Maintenance
Feasibility of implementation for providers	Once at end of implementation	Average score among providers on the Feasibility of Intervention Measure tool (FIM); qualitative interviews	Implementation

RE-AIM: Reach, Effectiveness, Adoption, Implementation, Maintenance; PrEP: Pre-exposure Prophylaxis; AIM: Acceptability of Intervention Measure; IAM: Intervention Appropriate Measure; PSAT: Program Sustainability Assessment Tool; FIM: Feasibility of Intervention Measure

### Data management, quality assurance, and ethical considerations

Data use agreements (DUAs) have been executed between relevant institutions so that investigators can share data related to implementation outcomes. Using an honest broker at each CHC, we have established a process to de-identify datasets, including transformation of dates of service and assignment of unique study identification codes (IDs) for patients. The business associate agreements (BAAs) and DUAs that have been executed between the partners involved in this study do not allow for the publication of anonymized data. Data sets will contain both individual-level data, including some limited health information that cannot be anonymized, and aggregate data. Thus, the data sets cannot be made publicly available.

Only minimally necessary data will be collected in both the pre-implementation and implementation phases. All data will be stored on secure servers at the University of Chicago. Only individuals with IRB approval and a record of human subject research training will have access to data during this study. Data will be stored until all dissemination activities for this study have been completed. Data will be transmitted to the University of Chicago using secure encrypted channels. Any data sent from the University of Chicago to other institutions will be de-identified.

We will establish a Data Safety Monitoring Board (DSMB) to protect the safety of study subjects and assure the quality of the research data generated is acceptable. The DSMB will consist of one chair and four members at the University of Chicago, Northwestern University, and other peer institutions. The Chair will convene the DSMB semi-annually for the duration of the program’s implementation to monitor data acquisition (accuracy, timeliness, and completeness) and issues related to participant safety and confidentiality, as well as to assess the progress of the study and the quality of data management and analysis. We will employ established procedures for adverse event (AE) reporting at the University of Chicago. The DSMB will have access to interim analyses/results and provide input on stopping guidelines.

This study is approved by the University of Chicago Institutional Review Board (IRB 21–0971). All organizations that are considered engaged in research for this study have agreed to rely on the IRB at the University of Chicago. Informed verbal consent will be obtained from participants for the pre-implementation focus groups and for the quantitative surveys and the individual interviews conducted as part of evaluating the implementation of POWER Up. The extraction of patient data from the EMR during the implementation period is considered part of routine care and does not require explicit consent from participants. DUAs and BAAs have been executed between institutions for the sharing of safe harbor and limited datasets for patients.

### Modifications to protocol

The team will continue to modify the protocol as needed to strengthen the study design and facilitate implementation and evaluation of the POWER Up strategies at each participating site. All proposed changes will be discussed with implementation partners and key stakeholders and will be approved by the University of Chicago IRB prior to application. Notification of amendment approvals and subsequent protocol modifications will be disseminated to the appropriate parties.

### Analysis

#### Qualitative data

For focus groups and individual interviews conducted during the pre-implementation, implementation, and post-implementation phases of the study, rapid qualitative analysis (RQA) and thematic coding will be utilized [38–40]. The research team will create a codebook with unique definitions for each code based on the CFIR [28], and Dedoose software will be used for analysis [41]. During the pre-implementation phase, qualitative data will be utilized to inform the adaptation of the POWER Up strategies, taking specific clinic and contextual factors into consideration.

During the implementation and post-implementation phases, we will conduct individual interviews with providers and patients. We will assess the process of implementing the POWER Up strategies and gather feedback on the strengths and weaknesses of the implementation via interview guides based on the CFIR. We will analyze these data using Dedoose to elicit key themes that will inform how we improve the process of implementing the bundle of POWER Up strategies at other sites nationwide. Additionally, we will document how the POWER Up strategies are adapted and modified at each clinic during the implementation process using the Framework for Reporting Adaptations and Modifications to Evidence-based Implementation Strategies (FRAME-IS) [42]. These findings will be included in our dissemination guide to help illustrate significant processes and mechanisms by which the bundle of POWER Up strategies exerted its effects.

#### Quantitative data

For EMR variables, we will use descriptive statistics to examine the distribution of the data. We will compare the distribution of patient characteristics between the unexposed observations from the control period and the exposed observations during the implementation period. Outcome rates will be plotted over each six-month time period, comparing how rates differ from the control period to the implementation period between CHCs within a geographic area (Midwest or South), as well as within each CHC, to examine the effects of the implementation site and calendar time on outcomes. Additionally, rates will be compared by geographic area to detect differences in implementation effect size between the Midwestern and the Southern sites. We will use an intention-to-treat analysis to ensure that if a CHC deviates from the assigned implementation timeline (i.e., they begin the implementation of the bundle of POWER Up strategies earlier or later than planned), the most conservative estimate of the size of the implementation is used. To examine treatment effects for the effectiveness and individual maintenance outcome related to PrEP uptake (initial prescriptions and refills), we will use a generalized linear mixed model, with a random effect for each CHC and a random effect of time per CHC [35, 43–45].

For RE-AIM outcomes, we will first use descriptive statistics to examine the distribution of the data. Bivariate analysis will be employed to explore differences between groups (e.g., differences between clinics or time points). Fisher’s exact test and chi-square tests (for categorical variables) and t-tests (for continuous variables) will be used to identify factors correlated with RE-AIM outcomes. All statistical analyses will use two-tail alpha to reject null hypotheses at 0.05. To measure associations of various factors with RE-AIM outcomes, we will report odds ratios along with 95% confidence intervals. Statistical assumptions such as data normality will be confirmed, or data transformations or alternate non-parametric methods that do not break model assumptions will be used. We anticipate nominal missing data, but we are willing to consider methods such as multiple imputation if needed to preserve sample size.

## Discussion

To effectively scale up our implementation strategies for use in CHCs, we need to monitor and evaluate each adaptation and modification step. Monitoring adaptation will ensure our strategies continue to support the overall model for our desired outcome of increased PrEP uptake and persistence among all patient populations, especially Black women. Successful implementation will begin with training of clinical staff followed by preliminary and setting-specific modifications, then adoption and evaluation of multilevel outcomes. As part of this project, we will complete multiple steps of iterative modification, implementation, and evaluation of our strategies to refine and test the POWER Up strategies for wide-scale use in CHCs in the Midwest and South. Our team will help facilitate provider training sessions, disseminate patient education materials, provide technical and logistical support for EMR updates, and serve as a resource for PrEP navigators and clinical champions. We will continually monitor the implementation process at each site to further adapt and support as needed. Lastly, we will measure the acceptability and fidelity of each strategy to gain additional feedback on the strengths and weaknesses of the strategy bundle and determine via EMR data if we achieved our goal (increased PrEP uptake).

Because our study involves multiple sites in diverse geographic regions and will involve a SW-CRT, we anticipate different challenges may arise over the three-year period of implementation and evaluation. Conducting a stepped-wedge trial can present some issues, particularly related to recruitment and implementation. There are often changes to the planned trial duration (either extension or shortening), delayed implementation due to logistical or technical issues (e.g., loss of funding/resources, staff turnover, or complete refusal of implementation), recruitment challenges (e.g., fewer participants enrolled than originally expected), and deviation from the planned analysis strategy [46]. To mitigate potential risks that could impact our proposed implementation schedule, we built several extra time periods into the design of this study. For instance, we included an entire year of pre-implementation study time to allow us to assess current PrEP workflows and processes at the sites, as well as to build trust and relationships with key personnel (e.g., clinical champions) at each CHC. We also allowed for a transition period for each CHC that will act as dedicated provider training periods and will also ensure sites have sufficient time to implement the bundle of POWER Up strategies before the actual implementation period begins. We have also created a tracking framework based on the FRAME-IS to record necessary adaptations for each strategy according to specific site needs. This form will allow us to illustrate any deviations and the subsequent changes that were made to our study design and/or methods.

Additionally, there are always potential challenges in studies with a diverse group of stakeholders who have varying experiences and competing priorities. For instance, the randomization of sites could cause some frustration amongst stakeholders. Some leaders may want certain sites to receive the bundle of implementation strategies before others due to financial/staff resources or perceived need in the community. Likewise, it may be difficult to recruit providers and get staff buy-in for the study at sites with a high volume of patients. However, we believe that having clinical champions at each site will alleviate some of these concerns and serve as a frequent reminder of the study’s importance and potential benefits for their patient population.

By focusing on the prevention of HIV infection among Black women in the Southern U.S., this study will address the EHE pillar to “Protect individuals at risk for HIV using evidence-based prevention approaches" [47]. Furthermore, developing an implementation guide for PrEP care continuum engagement and delivery among Black women will allow for replication of these strategies across EHE priority jurisdictions. This dissemination guide will provide the basis for broader implementation research studies of PrEP initiation and persistence within diverse settings. We will also disseminate results from this study to the participating sites via data and written reports and in the form of abstracts to conferences and publications to peer-reviewed journals. We believe this study is an important effort to reduce the gap in PrEP service delivery and increase PrEP uptake among a vulnerable population that is disproportionately affected by HIV. If proven to be an effective and acceptable bundle of implementation strategies, POWER Up could have a far-reaching impact on not only improving the PrEP rate among Southerners and Black women, but also eliminating HIV transmission nationwide.

## Trial status

The pre-implementation phase will end in June 2023 (i.e., the stepped-wedge trial is currently in the six-month control period). For the first three sites, the transition period of the implementation phase will begin in July 2023, with these three randomized CHCs receiving training and information on the bundle of POWER Up implementation strategies. These same three sites will begin their implementation period in January of 2024.The research team will modify procedures as needed to ensure successful implementation at each CHC.
